# Adult-onset vanishing white matter disease with the EIF2B2 gene mutation presenting as menometrorrhagia

**DOI:** 10.1186/s12883-019-1429-9

**Published:** 2019-08-22

**Authors:** Cuibai Wei, Qi Qin, Fei Chen, Aihong Zhou, Fen Wang, Xiumei Zuo, Rong Chen, Jihui Lyu, Jianping Jia

**Affiliations:** 1 Innovation center for neurological disorders, Department of Neurology, Xuan Wu Hospital, Capital Medical University, 45 Changchun Street, Beijing, 100053 China; 2 Center of Alzheimer’s Disease, Beijing Institute for Brain Disorders; Beijing Key Laboratory of Geriatric Cognitive Disorders, Neurodegenerative Laboratory of Ministry of Education of the People’s Republic of China, Beijing, China; 3 Department of Obstetrics and Gynecology, Peking Union Medical College Hospital, Peking Union Medical College, Chinese Academy of Medical Science, Beijing, China; 4 Center for Cognitive Disorders, Beijing Geriatric Hospital, Beijing, China

**Keywords:** Leukodystrophies, Vanishing white matter disease, Ovarioleukodystrophy, Eukaryotic translation initiation factor 2B, Adult-onset vanishing white matter disease, Late-onset vanishing white matter disease

## Abstract

**Background:**

Vanishing white matter disease (VWMD) is one of the most prevalent inherited leukoencephalopathies, which generally presents in childhood as a progressive disorder while less beginning in adulthood. The present report describes the clinical, neuroimaging, and genetic findings of a female patient with adult-onset VWMD. In addition, to provide a clearer delineation of the clinical and genetic characteristics of female adult-onset VWMD patients, 32 genetically confirmed female adult-onset EIF2B-mutated cases are summarized.

**Case presentation:**

The patient described here suffered from long-term menometrorrhagia prior to manifesting progressive neurological impairments that included tremors, bilateral pyramidal tract injury, cerebellar ataxia, and dementia. To the best of our knowledge, this is the first female patient with adult-onset VWMD suffering from long-term menometrorrhagia attributed to the c.254 T > A and c.496A > G mutations in the EIF2B2 gene; the c.496A > G mutation has not been reported in previous studies. The patient also exhibited metabolic dysfunction. The present findings widen the spectrum of phenotypic heterogeneity observed in VWMD patients.

**Conclusions:**

The present report summarizes 33 female patients with adult-onset VWMD to provide an overview of the clinical and genetic characteristics of this disorder and ovarioleukodystrophy. The mean age of clinical onset in female patients with adult-onset VWMD was 36.8 years and the neurological symptoms primarily included motor and cognitive dysfunction such as paraparesis, cerebellar ataxia, and executive deficits. In addition, ovarian failure occurred in all of these female patients and usually preceded the neurological symptoms. Furthermore, several patients also suffered from metabolic dysfunction. All 33 patients had mutations on EIF2B1–5, and of these, the c.338 G > A mutation in the EIF2B5 gene (p.Arg113His) was the most common. These findings suggest that clinicians should be aware of adult-onset forms of VWMD as well as its typical magnetic resonance imaging (MRI) and clinical characteristics although this pathology is usually recognized as a pediatric disorder. No curative treatment is presently available, and thus early recognition is important to prevent triggering events and to allow for genetic counseling.

**Electronic supplementary material:**

The online version of this article (10.1186/s12883-019-1429-9) contains supplementary material, which is available to authorized users.

## Background

Vanishing white matter disease (VWMD; OMIM 603896), which is also known as childhood ataxia with central nervous system hypomyelination (CACH), is an autosomal recessive leukoencephalopathy caused by mutations in the EIF2B1–5 genes that encode the subunits of eukaryotic translation initiation factor 2B (eIF2B) [1, 2]. eIF2B is indispensable for the initiation of translation and the regulation of protein synthesis under various conditions, including cell stress [3]. Characteristic neuropathological abnormalities in VWMD patients indicate that there are selective disruptions of oligodendrocytes and astrocytes whereas neurons are spared [4].

Although VWMD was initially recognized as a disorder of young children, it has become clear that there is extreme variation in disease phenotype and severity [5]. VWMD phenotypes range from a congenital form to an infantile form (onset age: 1 year) [6], an early childhood-onset form (onset age: 2–4 years) [5], a juvenile-onset form (onset age: 5–15 years) [7], and an adult-onset form (onset age: > 15 years) [8]. The severe forms of VWMD begin in the prenatal or early infantile period and manifest as cerebellar ataxia and spasticity and lead to early death [9]. Milder variants begin in adolescence or adulthood and are characterized by a slow disease progression [10]. The clinical signs in adults include seizures, spasticity, cerebellar syndrome, dementia, and manifestations of ovarian failure. Adult-onset VWMD occurring in association with primary ovarian failure is described as ovarioleukodystrophy [11], which is an extremely rare condition. Since it was first identified in 1996 [12], fewer than 30 genetically confirmed cases of ovarioleukodystrophy have been reported in the literature. Due to the small number of known cases, most clinicians have a relative lack of knowledge regarding the clinical presentation and neuroimaging features of VWMD ovarioleukodystrophy. Furthermore, there are many phenotypic variants of VWMD, which makes a definitive diagnosis difficult.

The present report describes a female patient with adult-onset VWMD who had two novel heterozygous mutations in the EIF2B2 gene. Distinct from other female cases that are frequently associated with premature ovarian failure, this is the first female patient with adult-onset VWMD suffering from long-term menometrorrhagia. To provide a clearer delineation of the clinical evaluation and genetic presentation of female patients with this disease, the relevant literature was reviewed. This report describes the wide range of phenotypic variants of VWMD and provides insight into early diagnostic strategies.

## Case presentation

A 25-year-old female presented with a 6-year history of long menstrual periods and a 4-year history of tremors in both hands. She showed clear signs of developmentally delayed intelligence in childhood. The neurological examination revealed a positive Babinski sign in the right lower limb and a slight decline in intellect (Wechsler Adult Intelligence Scale IQ score, IQ = 81), with particularly decreased cognitive scores on calculation and memory (Mini-mental State Examination, [MMSE] = 24/30). According to the physical examination, the patient was overweight (BMI 27.3 kg/m^2^), but no other abnormalities were found, including with regard to blood pressure, temperature, and pulse rate. She often felt tired and slept (cumulative sleep time 10–12 h/day). She had a slightly increased appetite, leading to four meals per day plus snacks. Her hands and feet sweat even in normal temperature. No similar symptoms were found in the patient’s relatives, and no head trauma or stress was reported.

**Fig. 1:**
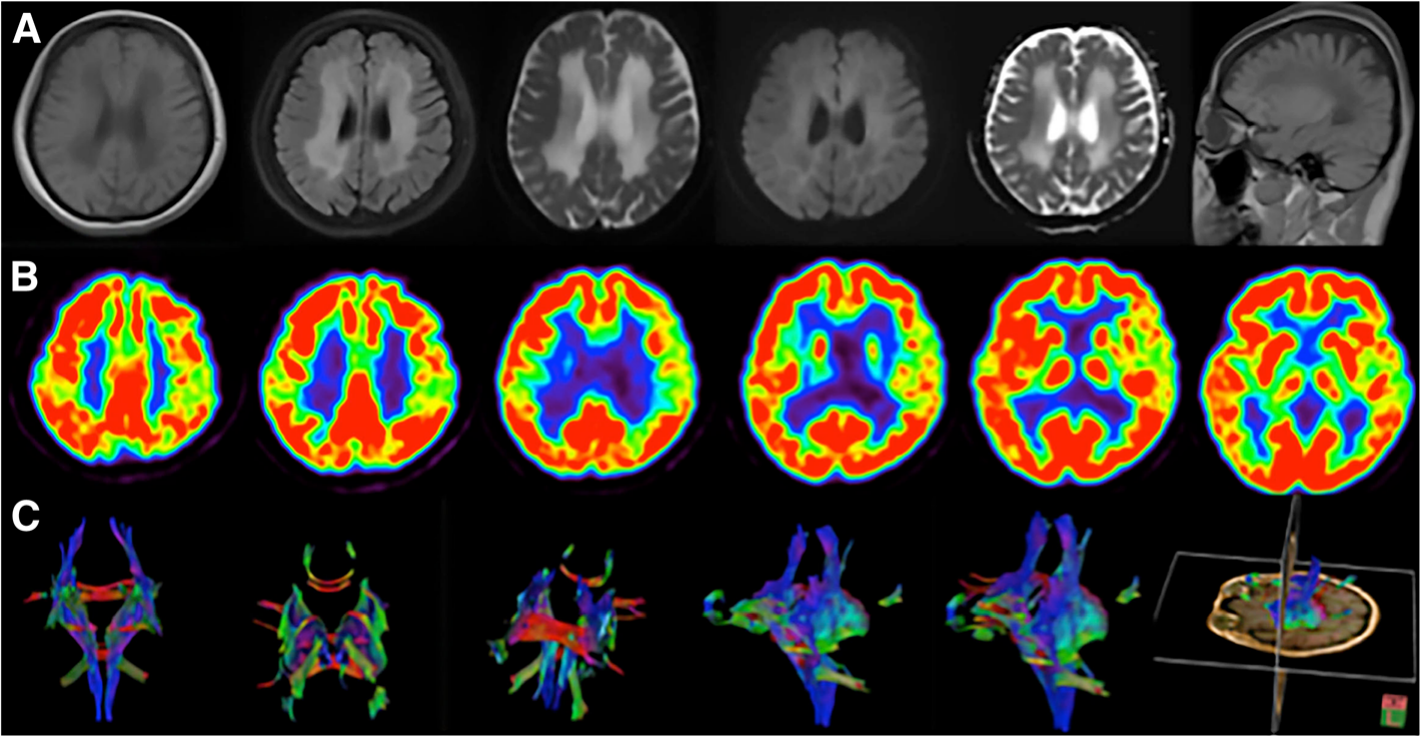
Neuroimaging results of the VWMD patient. **a** Axial T1-weighted, T2-weighted, Flair, DWI, ADC and coronal T1-weighted images showing extensive cerebral white matter abnormalities with central areas resembling signal intensity of cerebrospinal fluid surrounded by a rim of hyperintensity in the periventricular and subcortical regions. White-matter rarefaction and cystic degeneration are more evident in subcortical and periventricular regions. Obvious brain atrophy was also detected. **b** Brain ^18^F-FDG PET showed decreased FDG uptake in left frontal, parietal and temporal lobe. Compared with right side, the decline rate is 17, 12 and 17%. **c** Brain DTI disclosed fibrous white bundle was reduced sparsely among bilateral periventricular

She was first admitted as a gynecological outpatient for prolonged menstruation and subsequently transferred to our department due to an abnormal brain magnetic resonance image (MRI), which revealed diffuse and symmetric white matter abnormalities, with lesions having cerebrospinal fluid (CSF)-like signals (Fig. 1a). ^18^Flurodeoxyglucose positron emission tomography/computed tomography revealed decreased metabolism in the left frontal, parietal, and temporal lobes as well as in the cerebellum, and this may have been correlated with patient’s impaired calculation and memory deficits (Fig. 1b). Brain diffusion tensor imaging disclosed reduced fibrous white bundles among bilateral periventricular areas (Fig. 1c). Initial blood chemistry revealed only hypochromic microcytic anemia (hemoglobin, 82 g/L) and mild hypertriglyceridemia (triglycerides, 2.35 mmol/L) and hypoglycemia (glucose, 3.78 mmol/L). A cerebral spinal fluid (CSF) analyses were negative for encephalitis and paraneoplastic autoimmune encephalitis, and the patient had normal levels of myelin basic protein and aquaporin-4 with no neuromyelitis optica and a normal cell count without oligoclonal bands. A gynecological examination revealed normal levels of gonadal hormones, and an ultrasound revealed normal ovaries and uterus. A pathological examination of the endometrium showed both simple and complex hyperplasia based on a curettage surgery due to long duration menstruation and a large volume of menstrual blood in the patient.

Targeted sequencing performed on proband to screen pathogenic mutations. PCR combined Sanger sequencing then performed on three family members including one affected and two unaffected individuals to determine whether mutations cosegregate with disease. Blood samples were collected and stored in 4 °C until analysis. DNA isolation and high-throughput sequencing Genomic DNA was extracted from peripheral blood according to manufacturer’s instructions (QIAGEN, Hilden, Germany). DNA sequencing libraries were then prepared as followed according to Illumina standard protocol: genomic DNA was fragmented; Illumina adapters were ligated to the fragments after ‘A’ ligating to their 3’ends; Fragments with sample size in 200 to 500 base pair were selected and amplified by PCR (each sample is tagged with a unique index during this procedure). Fragments in the exonic regions of targeted genes were captured by a specific Hereditary Leukoencephalopathies Disease GenePanel using biotinylatedoligo-probes (MyGenostics GenCap Enrichment Technologies, MyGenostics, Baltimore, MD, USA). The Panel was designed to detect the coding region of 165 genes which cover almost all of genes that reported to relate to hereditary leukoencephalopathies disease (Additional file 1: Table S1). The capture experiment was conducted according to the manufacturer’s protocol. Briefly, 1 μg DNA library was mixed with Buffer BL and GenCap gene panel probe. The mixture was heated at 95 °C for 7 min and 65 °C for 2 min. Adding 23 μl of the 65 °C prewarmed Buffer HY (MyGenostics) to mixture, then hybridizate at 65 °C for 22 h. After adding 64 μl 2X binding buffer and 80 μl MyOnebeads (Life Technology), the hybrid mixture was transferred to the tube. The mixture was rotated and the beads were washed. The bound DNA was then eluted followed by amplification activated at 98 °C for 30 s (1 cycle), 98 °C for 25 s, 65 °C for 30s, 72 °C for 30s (15 cycles), 72 °C for 5 min (1 cycle). The PCR products were purified and then sequenced by Illumina HiSeq Tm 2000 sequencer, generating 2 × 100 bp reads. Base was called using the Off-Line Base Caller v1.9.

**Fig. 2:**
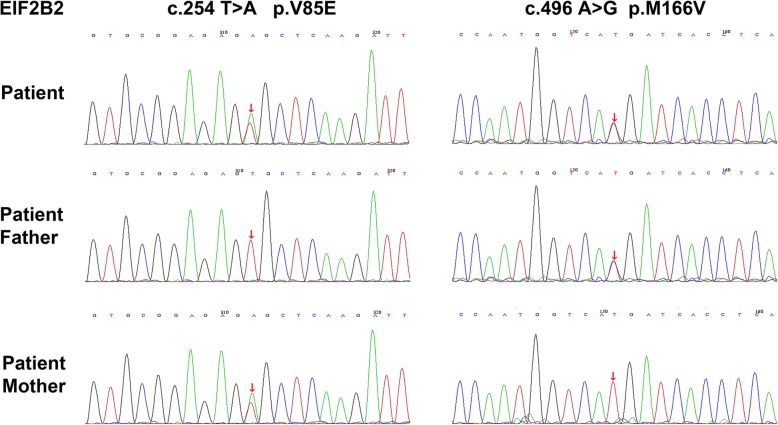
Genetic results of the VWMD patient. Genomic sequence chromatograms of the missense in EIF2B2 in patient’s family members: c.254 T > A and c.496A > G

We diagnosed vanishing white matter disease (VWMD) based on compound heterozygosis for the EIF2B2 gene mutation and the diffuse symmetric abnormality in white matter Fig. 2.

A 10-month follow-up examination showed a mild decline in intelligence (IQ decreased from 81 to 73), a slightly aggravated pyramidal tract (positive bilateral Babinski and Chaddock signs), and light cerebellar ataxia (unsteady bilateral knee-shin and finger-nose signs). The tremors in the hands had not changed, and she still demonstrated a prolonged menstrual period with an irregular menstrual cycle 3 months after the curettage. A brain MRI showed an image coincident with the prior one. Written informed consent was obtained from the patient prior to publication of this report.

## Discussion and conclusions

### Review of the literature

Prior to this report, 32 cases of female patients with adult-onset VWMD had been published. To provide an overview of the clinical characteristics of female patients with adult-onset VWMD and ovarioleukodystrophy, searches of the PubMed and MEDLINE databases were performed in March 2019 using the terms “adult onset vanishing white matter disease,” “late onset vanishing white matter disease,” “eukaryotic translation initiation factor 2B,” or “ovarioleukodystrophy”; all identified articles published in English and articles referenced therein were reviewed. The patient inclusion criteria consisted of: disease onset after the age of 15 years, mutation in one of the EIF2B1–5 genes, and being female.

In addition to the present case, a total of 32 genetically confirmed cases were identified and summarized to provide an overview of the clinical and genetic features of female adult-onset VWMD (Table 1).

**Table 1:** Clinical and genetic evaluation of the female adult onset VWMD patients

Patient Number/ Reference	Age onset (years)	Neurological Presentation			Ovarian Function	Metabolic disturbance	Mutated Gene in EIF2B	Mutation on Genomic DNA	Amino Acid Change
		Motor Dysfunction	Cognitive Dysfunction	Other Symptoms					
1 [11, 12]	23	Gait instability Bilateral Babinski sign	IQ = 7 Visuo-spatial deficits	–	Primary amenorrhea	–	EIF2B4	C1393T C1465T	C465R Y489H
2 [11, 12]	24	Facial-oral-hand apraxia	IQ = 52 Frontal lobe deficits	Seizures	Delayed menarche	Obesity Hyperlipidemia	EIF2B4	C1393T C1465T	C465R Y489H
3 [11, 12]	29	Cerebellar and pyramidal deficits	Normal IQ	Dysarthria	Primary amenorrhea	–	EIF2B2	C547T A638G	R183stopE213G
4 [11]	33	Gait instability	IQ = 60 Executive deficits	–	Secondary amenorrhea at 26	–	EIF2B2	C512T 60712del/insTG	S171F M203 fs
5 [11]	16	–	IQ not evaluated	Delayed speech	–	–	EIF2B2	C547T A638G	R183stopE213G
6 [11]	31	Gait instability	IQ not evaluated	Anxiety and depression	Secondary amenorrhea at 27		EIF2B5	G338A	R113H
7 [11]	48	Gait instability	MMSE = 21/30	Seizures	Secondary amenorrhea at 31	–	EIF2B5	G338A	R113H
8 [11]	16	Gait instability	IQ = 96	–	Primary amenorrhea	–	EIF2B5	G338A C538T	R113H R195C
9 [13]	46	Gait slowness	MMSE = 17/30’ Executive deficits	Apathy	–	–	EIF2B5	C545T C1340T	T182 M S447 L
10 [14, 15]	29	–	–	Aphasia Paresthesias	Secondary Amenorrhea at 20	All metabolite reduction	EIF2B3	C260T G272A	A87V A91H
11 [16]	56	Hands clumsiness Gait instability	Memory deficits	–	Secondary amenorrhea at 37	–	EIF2B2	T254A	V85E
12 [16]	53	Gait instability	Miscalculation	–	Secondary amenorrhea at 28.	–	EIF2B5	G808C	D270H
13 [16]	30	Left leg weakness	–	Left hemianopia	–	–	EIF2B3	T80A	L27Q
14 [17]	17	Gait slowness Cerebellar ataxia	IQ = 73	–	Secondary amenorrhea at 32	Hypogonadism	EIF2B2	A638G	E213G
15 [18]	66	Spastic paraparesis Brisk reflexes Babinski sign	Visuo-spatial memory deficits	–	Premature amenorrhea	–	EIF2B3	C260T	A87V
16 [19]	61	Incoordination Spastic paraparesis	IQ = 66	Seizures	–	–	EIF2B1	T715G	F239 V
17 [20]	17	Tremors Paresis	Mild cognitive deficits	Dysarthria paresthesia	–	–	EIF2B5	G338A	R113H
18 [21]	52	Spastic gait Brisk tendon reflexes	IQ = 51 MMSE = 15/30 Memory deficits	Emotional instability	Secondary amenorrhea at 31	Obesity Hyperlipidemia	EIF2B5	C545T	T182 M
19 [22, 23]	35	Cerebellar ataxia	MMSE = 23/30	–	Primary amenorrhea	Obesity Hyperlipidemia	EIF2B5	G338A	R113H
20 [22–24]	33	Pyramid dysfunction	–	Seizures	Secondary amenorrhea at 30	–	EIF2B5	G338A	R113H
21 [25]	23	–	–	Seizures	Infertility	Obesity Hyperlipidemia	EIF2B2	A638G A818G	E213G K273H
22 [22, 23]	52	Cerebellar ataxia	MMSE = 25/30	Seizures	–	Hyperlipidemia	EIF2B5	G338A	R113H
23 [22, 23]	55	Spastic paraparesis	–	–	Delayed menarche	–	EIF2B5	G338A	R113H
24 [22, 23]	37	Spastic paraparesis Cerebellar ataxia	–	–	Primary amenorrhea	–	EIF2B5	G338A	R113H
25 [22, 23]	44	Spastic paraparesis	MMSE = 27/30	–	–	–	EIF2B5	G584A A1448G	A195H T483C
26 [22, 23]	41	Spastic paraparesis Cerebellar ataxia	MMSE = 21/30	Seizures	–	–	EIF2B5	A641G C805T	H214A A269X
27 [22, 23]	38	Tremors Paresis	MMSE = 10/30	Seizures	Secondary amenorrhea at 35	–	EIF2B5	G338A	R113H
28 [22, 23]	39	Spastic paraparesis	MMSE = 4/30	Seizures	Secondary amenorrhea at 21	–	EIF2B5	G338A	R113H
29 [22, 23]	39	Cerebellar ataxia	–	–	Secondary amenorrhea at 18	–	EIF2B5	G338A	R113H
30 [26]	19	–	–	Seizures paresthesia	Primary amenorrhea	Hypoglycemia	EIF2B5	C869A A913T	A299H M305 L
31 [27]	59	Spastic paraparesis Babinski sign	IQ = 60 MMSE = 16/30	–	–	–	EIF2B4	T617C A952G	M206 T I318V
32 [28]	30	Weakness and spastic in left limb	Normal IQ and MMSE	–	Secondary amenorrhea at 25	–	EIF2B4	–	–
33 (the present patient)	25	Tremors Babinski sign	IQ = 81MMSE = 24 Miscalculation Memory deficits	–	Menometrorrhagia	Obesity, hypoglycemia, hypertriglyceridemia	EIF2B2	T254A A496G	V85E M166 V

### Neurological evaluation

The mean age of onset of female patients with adult-onset VWMD was 36.8 years (range: 16–66 years, standard deviation [SD]: 14.3). Motor dysfunction was detected in 29 patients (87.8%) and gait instability, spastic paraparesis, and cerebellar ataxia were the three main VWMD symptoms. More specifically, of the 33 patients, 11 (30%) suffered from spastic paraparesis, 9 (27.2%) exhibited gait instability, and 6 (18.1%) showed cerebellar ataxia. Tremors and weakness of the limbs were also evident in the patients.

Of the 33 patients, 25 (75.8%) had available data about cognitive function. Cognition was impaired in 20 patients (80%; mean MMSE score: 19.5, range: 4–27, SD: 6.89) and executive and memory deficits were the major signs of cognitive dysfunction. IQ tests (mean score: 65, SD: 11.2) revealed declines in intellect in eight patients.

Seizures occurred in 10 patients (30.3%) and paresthesias and psychiatric symptoms also caused deterioration in the VWMD patients.

### Evaluation of ovarian function

Of the 33 patients, 24 (72.7%) exhibited ovarian failure, 13 (54.2%) had secondary amenorrhea (mean onset age: 27.8 years, range: 21–37, SD: 5.73), 7 (29.2%) suffered primary amenorrhea, 3 (9.1%) presented with delayed menarche, and 1 suffered infertility; ovarian failure always preceded the neurological symptoms. In contrast to all previously identified cases, the present patient was the first to report suffering from long-term menometrorrhagia.

### Evaluation of metabolic dysfunction

Of the 33 patients in the present report, 5 (15.1%) were obese, 6 (18.2%) exhibited hyperlipemia, and 2 showed changes in glucose metabolism that presented as hypoglycemia.

### Genetic findings

All of the female VWMD patients included in the present report had their diagnoses genetically confirmed: 18 (54.5%) exhibited mutations in the EIF2B5 gene, 7 (21.2%) had them in the EIF2B2 gene, and 4 (12.1%) had them in the EIF2B4 gene. The recurrent c.338 G > A mutation in the EIF2B5 gene (p.Arg113His) was found in 12 patients (36.4%), including in 11 patients in a homozygous state. In the only case of a compound heterozygote for this recurrent mutation, the second mutation was the c.538 C > T. EIF2B2 gene, which is the second most common gene mutation in VWMD patients. The patient described in the present report was also confirmed to be carrying a compound heterozygote for the EIF2B2 gene mutations: c.254 T > A and c.496A > G. The c.254 T > A mutation in the EIF2B2 gene has been reported in female patients with adult-onset VWMD but, to the best of our knowledge, the c.496A > G missense mutation in the EIF2B2 gene has never been reported. All reported cases of the EIF2B4 mutations were heterozygous. Mutations in the EIF2B3 and EIF2B1 genes are relatively rare and only one patient was confirmed to be carrying an EIF2B1 gene mutation.

The present findings indicate that VWMD presents along a wider clinical spectrum than previously thought. The mean age of clinical onset of the female adult-onset VWMD patients in the present report was 36.8 years old and the related neurological symptoms primarily involved motor and cognitive dysfunction such as paraparesis, cerebellar ataxia, and executive deficits. Ovarian failure occurred in all female VWMD patients and usually preceded the neurological symptoms. In addition, several patients suffered from physical dysfunction. All of the female adult-onset VWMD patients had mutations on EIF2B1–5, and of these, the c.338 G > A mutation in the EIF2B5 gene (p.Arg113His) was the most common.

## Discussion

VWMD generally presents in childhood as a progressive disorder with episodes of rapid and major neurological deterioration induced by stressors [29]. Much milder variants begin in adulthood and are characterized by slowly progressive spastic paraparesis and cerebellar ataxia. Ovarian dysfunction is frequently reported in females with VWMD, particularly those with abnormal menstruation, and can precede the neurological symptoms [30]. This condition is known as ovarioleukodystrophy, which is an extremely rare condition as fewer than 30 genetically confirmed cases have been reported. The present report summarized genetically confirmed cases in the literature to provide an overview of the clinical and genetic features of adult-onset female VWMD. To the best of our knowledge, this case series is the largest ever reported and one of the first to describe a female adult-onset VWMD patient suffering from progressive neurological impairment, including tremors, bilateral pyramidal tract injury, cerebellar ataxia, and dementia. Prior to the appearance of these neurological symptoms, this patient showed long-term menometrorrhagia. This case was distinctly different from previous cases, as the ovarian function and morphological structure of our patient were normal. The present patient also exhibited physical symptoms due to systemic injuries that included metabolic dysfunction (obesity, hypertriglyceridemia, and hypoglycemia), abnormal feeding behaviors (more frequent eating), and autonomic nervous dysfunction (sweating of the limbs). Based on these clinical manifestations, it is possible that multi-system injuries were involved in the pathogenesis of this case of VWMD.

**Fig. 3:**
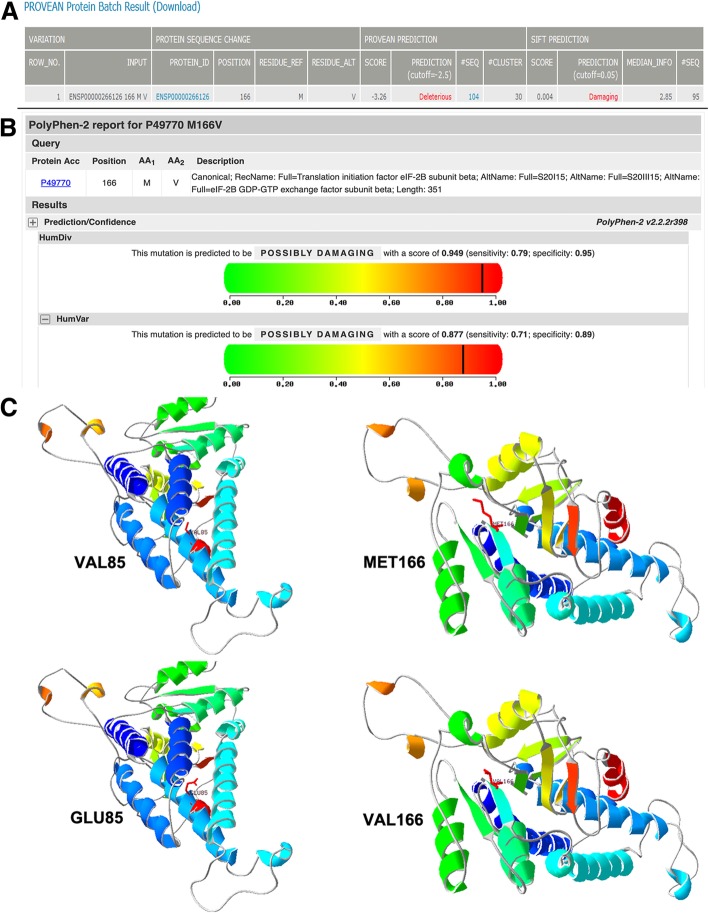
Function prediction and protein molecular models: **a** SIFT and **b** POLYPHEN2 online websites were used to analyze the amino acid substitutions of p.M166 V. Both programs predicted the mutation c.496A > G to be deleterious, which means they probably damage and affect protein functions. **c** The mutation protein E85 has a different side chain to the wild-type protein V85. The wide-type protein has a longer side chain than the mutation V166 protein

VWMD is an autosomal recessive leukoencephalopathy caused by mutations in any of five genes (EIF2B1–5) encoding the eIF2B subunits, which is a protein complex involved in the regulation of the first step of protein synthesis [31]. Activity of ElF2B guanine nucleotide exchange factor is subject to direct control through its own phosphorylation, which leads to a rapid reduction in protein synthesis [32]. Neuropathological abnormalities in patients with VWMD indicate selective disruption of oligodendrocytes and astrocytes with sparing of neurons. However, how the gene mutation causes nerve injury is unknown. Until now, more than 120 mutations in EIF2B1–5 have been identified [33]. Our patient was confirmed to carry a compound heterozygote for the EIF2B2 gene mutations c.254 T > A and c.496A > G. The c.254 T > A mutation in the EIF2B2 gene has been presented three times (Table 2) [16, 34], including one female adult-onset female with VWMD [35]. The c.254 T > A missense mutation leads to a single substitution within coding region from thymine to adenine, resulting from valine to glutamic acid (p.V85E). The function of mutant c.254 T > A was predicted to be pathological and had been reported in the Human Gene Mutation Database (HGMD) database (http://www.hgmd.cf.ac.uk/ac/index.php). In 2011, Matsukawa revealed that the mutant c.254 T > A subunit could cause approximately 20% decreased GDP/GTP exchange activity of eIF2B [16]. To the best of our knowledge, the c.496A > G missense mutation in the EIF2B2 gene has never been reported. It causes adenine change to guanine, resulting from methionine to valine (p.M166 V). This mutation does not belong to polymorphism sites and has never been found in the population. SIFT (http://sift.jcvi.org/) and POLYPHEN2 (http://genetics.bwh.harvard.edu/pph2/) were used to analyze the amino acid substitutions of p.M166 V (Fig. 3a and b). Both programs predicted the mutation c.496A > G to be deleterious, which means they probably damage and affect protein functions. Furthermore, protein tertiary structures were modeled with SWISS-MODEL (http://www.swissmodel.expasy.or-g/), which predict the sequence homology. The p.V85E protein model revealed that the mutation c.254 T > A lead to the amino acid side chain change through the substitution of valine to glutamic acid. The p.M166 V protein model predicted that the mutation c.496A > G affected the amino acid side chain by the substitution of methionine to valine (Fig. 3c).

**Table 2:** Reported cases with c.254 G > A mutation in the EIF2B2 gene

Patient Number/ Reference	Onset phenotype	Mutated Gene in EIF2B	Mutation on Genomic DNA	Amino Acid Change
1 [34]	Infantile	EIF2B2	T254A G922A	V85G V308 M
2 [35]	Early childhood	EIF2B2	T254A C995T	V85G A332V
3 [16]	Adult onset	EIF2B2	T254A	V85G
4 (the present patient)	Adult onset	EIF2B2	T254A A496G	V85G M166 V

Brain MRI scans of VWMD patients typically show diffuse symmetric abnormalities of the cerebral white matter with the lesions exhibiting characteristic cerebrospinal fluid (CSF)-like signals [36]. These white matter hyperintensities are followed by cerebral white matter rarefaction and cystic degeneration in which the white matter is replaced by fluid [37].

## Conclusions

In conclusion, the present report described the clinical, neuroimaging, and genetic findings of a female adult-onset patient with VWMD. To the best of our knowledge, this is the first female adult-onset patient with VWMD suffering from long-term menometrorrhagia due to novel EIF2B2 gene mutations. Furthermore, the cases of 33 female patients with adult-onset VWMD were summarized to provide an overview of the clinical and genetic characteristics of this disorder and ovarioleukodystrophy. Our description enlarges the spectrum of phenotypic heterogeneity in VWMD. Clinicians should be aware of adult and milder forms of VWMD, although VWMD is usually recognized as a pediatric disorder. No curative treatment is currently available. Thus, early recognition is important to prevent triggering events and to allow for genetic counseling.

## Additional file

Additional file 1: **Table S1.** Genes detected relate to hereditary leukoencephalopathies disease. (DOCX 18 kb)

## Data Availability

All data generated or analyzed during this study are included in this published article.

## Abbreviations

CACH: Central nervous system hypomyelination
CSF: Cerebrospinal fluid
eIF2B: eukaryotic translation initiation factor 2B
IQ: Wechsler Adult Intelligence Scale IQ score
MMSE: Mini-mental State Examination
MRI: Magnetic resonance image
SD: Standard Deviation
VWMD: Vanishing white matter disease
