# Supplementary material for: Adult-onset vanishing white matter disease with the EIF2B2 gene mutation presenting as menometrorrhagia
Source: BMC Neurol. 2019 Aug 22;19:203. doi: 10.1186/s12883-019-1429-9 (PMC6704498; doi:10.1186/s12883-019-1429-9)
Supplement: Supplementary file 1 — Table S1. Genes detected relate to hereditary leukoencephalopathies disease. (DOCX 18 kb) [file 12883_2019_1429_MOESM1_ESM.docx]

| AARS2 | CYP27A1 | ETHE1 | HEPACAM | NDUFA10 | NOTCH3 | PEX7 | SDHAF1 |
| --- | --- | --- | --- | --- | --- | --- | --- |
| ABAT | DARS2 | FA2H | HFE | NDUFA11 | NR0B1 | PHYH | SLC16A2 |
| ABCD1 | DBT | FAM126A | HSPD1 | NDUFA12 | NUBPL | PINK1 | SLC17A5 |
| ACOX1 | DDC | FASTKD2 | HTRA1 | NDUFA2 | OCRL | PLA2G6 | SLC20A2 |
| ACTA2 | DLD | FKTN | HTRA2 | NDUFA9 | PARK2 | PLAU | SNCA |
| ADH1C | DNAJC13 | FOLR1 | L2HGDH | NDUFAF1 | PARK7 | PLP1 | SOX10 |
| AIMP1 | DNAJC6 | FOXRED1 | LMBRD1 | NDUFAF2 | PAXIP1 | POLR3A | SUMF1 |
| ALDH3A2 | ECM1 | FUCA1 | LMNB1 | NDUFAF3 | PC | POLR3B | SURF1 |
| APBB2 | EIF2B1 | GABRG2 | MBP | NDUFAF4 | PDGFRB | PSAP | TBP |
| ARSA | EIF2B2 | GALC | MCCC1 | NDUFAF6 | PEX1 | PSEN2 | TREM2 |
| ASPA | EIF2B3 | GALE | MGP | NDUFB3 | PEX10 | PTCH1 | TREX1 |
| BCKDHA | EIF2B4 | GALK1 | MLC1 | NDUFS1 | PEX12 | PTCH2 | TUBB4A |
| BCKDHB | EIF2B5 | GALT | MMAA | NDUFS2 | PEX13 | RASA1 | TUFM |
| BCS1L | EIF4G1 | GAN | MMAB | NDUFS3 | PEX14 | RNASEH2A | TYMP |
| CHM | ERCC2 | GBA | MMACHC | NDUFS4 | PEX16 | RNASEH2B | TYROBP |
| CLCN2 | ERCC3 | GFAP | MMADHC | NDUFS6 | PEX19 | RNASEH2C | UCHL1 |
| COA5 | ERCC6 | GIGYF2 | MPV17 | NDUFS7 | PEX2 | RNASET2 | ATP7B |
| COX15 | ERCC8 | GJA1 | MPZ | NDUFS8 | PEX26 | RPIA | ATP7A |
| COX6B1 | ETFA | GJB1 | MTRR | NDUFV1 | PEX3 | SAMHD1 |  |
| CSF1R | ETFB | GJC2 | MUT | NDUFV2 | PEX5 | SCP2 |  |
| CTC1 | ETFDH | GLB1 | NDUFA1 | NOS3 | PEX6 | SDHA |  |

Additional file 1: Table S1 Genes detected relate to hereditary leukoencephalopathies disease
